# Supplementary figures and images for: Pathogenesis of Port-Wine Stains: Directions for Future Therapies
Source: Int J Mol Sci. 2022 Oct 12;23(20):12139. doi: 10.3390/ijms232012139 (PMC9603382; doi:10.3390/ijms232012139)

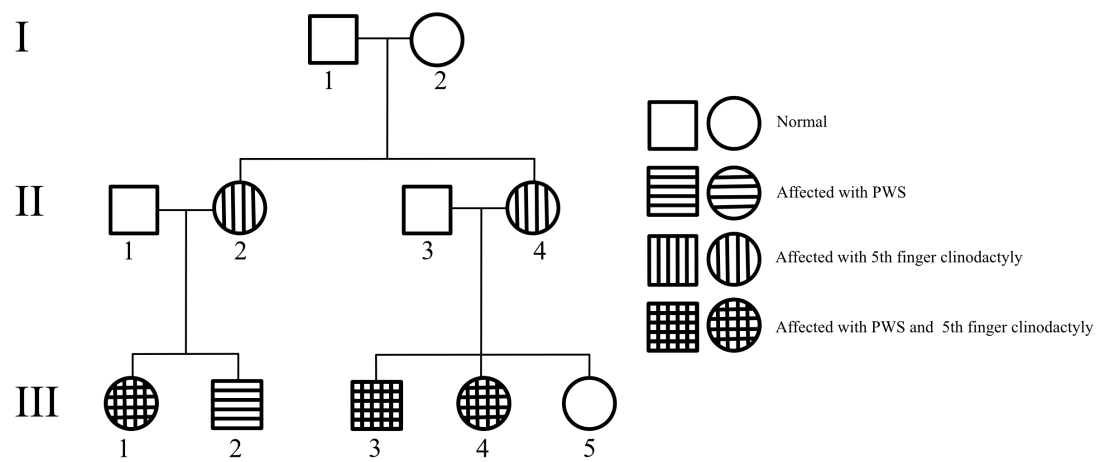

Figure S1 Family pedigree from the article “A family with hereditary port wine stain”

Supplement: Supplementary file 1 [file ijms-23-12139-s001.zip › ijms-1932403-supplementary.pdf]
